# Accuracy of clinical diagnosis and malaria rapid diagnostic test and its influence on the management of children with fever under reduced malaria burden in Misungwi district, Mwanza Tanzania

**DOI:** 10.11604/pamj.2016.25.48.9401

**Published:** 2016-09-29

**Authors:** Daniel Ndaki Nkonya, Donath Samuel Tarimo, Rogath Saika Kishimba

**Affiliations:** 1 Tanzania Field Epidemiology and Laboratory Training Program; 2 Department of Parasitology, School of Public Health & Social Sciences, Muhimbili University of Health & Allied Sciences, Dar es Salaam, Tanzania; 3 Tanzania Ministry of Health, Community Development, Gender, Elderly and Children

**Keywords:** Underfives, malaria, clinical diagnosis, mRDT, diagnostic accuracy, case management, Tanzania

## Abstract

**Introduction:**

Malaria diagnosis is known to be non-specific because of the overlap of symptoms of malaria with other infectious diseases that is made worse with declining malaria burden. Though the use of malaria rapid diagnostic test (mRDT) for malaria confirmation has universally been adopted, malaria decline may alter performance of mRDT. This study examined accuracy of clinical diagnosis and mRDT and its influence on prescription for febrile underfives.

**Methods:**

A cross-sectional study of 600 underfives was carried out in 6 randomly selected health facilities in Misungwi district, Mwanza; from November - December 2014. Consecutive underfives with a fever consultation were recruited: for each fever and the clinical diagnosis entertained were recorded. Parasitological confirmation of malaria was done by mRDT and microscopic examination of finger prick blood samples. Treatment was based on mRDT results, drugs prescribed recorded. Accuracy of clinical diagnosis and mRDT in predicting malaria was assessed by performance indices against microscopy. Antimalarial and antibiotics prescriptions were assessed against parasitological findings.

**Results:**

Clinically, 37.2% had malaria; 32.8% were mRDTpositive and 17.0% microscopically positive. Sensitivity of clinical diagnosis was very high (97.0% [95%CI: 91.0-99.2]); specificity 66.7% [95%CI: 62.3-70.8], and positive predictive value 37.4% (95%CI: 31.6-43.5). Sensitivity of mRDTwas very high (99.0% [95%CI: 93.9-99.9]), specificity (80.7% [95%CI: 76.9-84.0]), positive predictive value 51.3% [95% CI: 44.1-58.4]) and negative predictive 99.75% [95%CI: 99.4-100.0]. Those receiving antimalarial prescription, 75.0% were mRDT positive; 39.4% microscopically positive. Those receiving antibiotic, 78.8% were mRDT negative; 90.1% microscopically negative.

**Conclusion:**

Decline in malaria lowered specificity of mRDT to < 95% against WHO recommendation. Though adherence to mRDT results was high, there was over prescription of antibiotics.

## Introduction

Fever (raised body temperature) has been the entry point for the clinical diagnosis of malaria for decades; thus the first suspicion of malarial illness in an individual is based on the history of fever and / or measured fever [1]. Despite the decline in malaria burden in sub-Saharan Africa [2], fever has remained a major cause of outpatient attendance in health facilities [3], thus overemphasizing the need for a parasitological confirmation of malaria among patients of all age groups with fever in line with the World Health Organization (WHO) recommendation [4]. In most health facilities in peripheral settings, the operationally feasible parasitological confirmation of malaria is by use of malaria rapid diagnostic test (mRDT) [5]. In Tanzania, available data show that malaria incidence and prevalence have declined in most parts of the country as shown in the national survey of 2007/8 whereby the overall prevalence of malaria among underfives was 18.1%, while in the 2011/12 survey the overall prevalence was 9.7% by mRDT and 4.2% by microscopy [6, 7]. Based on the surveys, the decline was recorded in all geographical zones; and in both surveys the Lake, Southern and Western zones had the highest prevalence while the Northern and Southern high land zones had the lowest prevalence. The decline in malaria parasites prevalence and the number of cases of malaria related fevers may conceivably lower the specificity and predictive value of the clinical diagnosis of malaria as well as the specificity and predictive value of mRDT for the prediction of malaria parasitaemia [8]. The decrease in malaria prevalence, and declining proportion of fevers due to malaria [2, 6], which is known to affect the accuracy (sensitivity, specificity and predictive values) of clinical diagnosis and rapid malaria diagnostic tests [8], primarily prompted us to conduct this study. The secondary objective was to examine the influence of laboratory findings in the guidance of case management of underfives with fever in terms of prescribing antimalarial drug for the mRDT positives and an antibiotic for the mRDT negatives suspected to have a bacterial infection [9, 10]. Findings from this study are envisaged to appraise on the influence of laboratory confirmation of malaria on the management of underfives with fever and provide an informed policy decision for improving management of fever in primary health facilities [11]. Here we report the accuracy of a clinical malaria diagnosis and mRDT for malaria diagnosis under reduced malaria burden and its influence in the management of children attending to health facilities on account of fever.

## Methods

**Study area:** The study was conducted in Misungwi district, Mwanza region, north-west Tanzania located at an altitude of 1,178m above sea level; with a region malaria prevalence of 18.6% by mRDT and 5.4% by microscopy [7]. The district has two annual rainy seasons, the long rains between February and May and the short rains between November and December; the dry and relatively hot season is from June to September. Malaria transmission is seasonal, with peaks in one to two months after the rains [12].

**Study design and population:** A facility based quantitative cross sectional study was conducted in 6 randomly selected health facilities of Misungwi district between November and December 2014 among children under-five years of age attending to the respective health facilities on account of fever.

**Sample size estimation:** The sample size was estimated based on the reported prevalence of malaria of 12.0% among under-fives in one of the districts in Mwanza [13]. The margin of error (ε) was taken to be 2% at 95% confidence interval. The Sample size was computed using the formula: n = z^2^ p(1-p)/ε^2^ or n = z^2^p(100-p)/ε^2^ Where: Z = level of confidence (1.96 for 95% confidence level) p = expected prevalence (=12%) ε = margin of error = 2% →N = 1.962 x 12(100 - 12) = 518 Adjustment for non-response: for a facility study it was expected that the response rate (R) would be than 90%. To adjust for non-response, a factor F = 1/R was multiplied by N to get an adjusted sample size. In this case, 1/R x N = 1/0.9 x 518 = 575 was the minimum sample size; in this study 600 participants were recruited.

**Figure 1:**
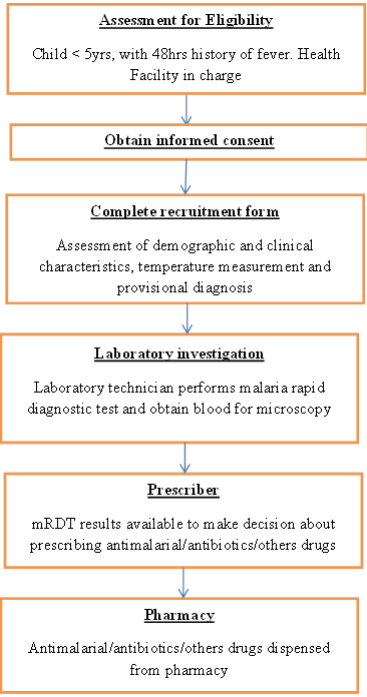
Patient flow in the study

**Sampling procedure:** To identify health facilities to be included in the study, a cluster sampling technique was used. This was carried out by classifying health facilities in three different clusters according to levels: hospital, health center or dispensary from which 6 facilities; a hospital, two health centers and three dispensaries were randomly selected. Thereafter the sample size allocated for each facility was based on the catchment population. Consecutive under-fives attending to the selected facilities on account of fever were recruited. A questionnaire was used to record the socio-demographic and clinical characteristics of the under-fives; for each a history of fever and axillary temperature (digital thermometry) were recorded. Parasitological malaria confirmation was done by mRDT and a prescription was given based on the mRDT findings. Blood smears for microscopic malaria confirmation were sent to the Parasitology laboratory at Muhimbili University of Health & Allied Sciences for expert microscopy. Two experienced microscopist unaware of the mRDT results performed microscopy each time comparing their results (Figure 1).

**Data Analysis:** Data were cleaned, entered and processed using SPSS computer software version 13. Analysis was carried out using SPSS version 13 whereby descriptive analyses were done by using frequencies and proportions to estimate magnitude of the outcomes of interest. Accuracy of clinical malaria diagnosis and mRDT for the identification of under-fives with malaria parasitaemia was assessed from the performance indices with their 95% CI. Influence of parasitological malaria confirmation on prescription of antimalarial and antibiotics was assessed against mRDT and microscopic findings.

**Ethical Consideration:** The study ethically cleared the Institutional Review Board of the Muhimbili University of Health and Allied Sciences. Informed consent for participation in the study was sought from parents/caregivers on behalf of the under-fives’. Administrative permission to carry out the study was sought from the appropriate Regional and District authorities as well as the Medical Officers in charge of all selected health facilities.

## Results

A total of 600 under-fives with a history of fever in the last 48 hours were referred to laboratory for parasitological confirmation of malaria by mRDT and microscopy. The characteristics of the study participants are shown in Table 1. Less than a quarter (22.3%) had received an antimalarial two weeks prior to the survey. On visit, less than a half (42.8%) had fever with a body temperature of 37.5οC-40.3οC; about a third (37.2%) had a clinical diagnosis of malaria.

**Table 1:** Characteristics of the study population (N = 600)

Attribute	No (%)
**Age (months)**	
1-12	268(44.7)
13-24	172 (28.7)
25-36	75 (12.5)
37-48	56 (9.3)
49-60	29 (4.8)
**Sex**	
Female	289(48.2)
Male	311(51.8)
**Facility Location**	
Urban	198 (33.0)
Rural	404 (67.0)
**Antimalarial treatment last 2 weeks**	
Yes	134(22.3)
No	466(77.7)
**Body temperature(˚C)**	
Febrile (37.5 – 40.3)	257(42.8)
Afebrile (36.0 – 37.4)	343 (57.2)
**Clinical Diagnosis entertained**	
Clinical Malaria	223(37.2)
Respiratory infection	238 (39.7)
Gastroenteritis	96 (16.0)
Other diagnoses	43 (7.2)

**Parasitological malaria diagnosis by mRDT and microscopy :** Of the 600 under-fives referred for parasitological malaria confirmation, 32.8% were positive by mRDT while only 17.0% were microscopically positive. Among those positive for malaria by microscopy, close to two thirds (62.7%) had parasite counts 10,000-350,000 / µL of blood (Table 2).

**Table 2:** Distribution of parasites counts among under-fives microscopically positive for malaria parasites (n =102)

Parasites density (counts / µL)	No (%)
1 - 1999	14 (13.73)
2000 - 9999	24 (23.53)
10000- 99999	56 (54.90)
100,000 -350,000	8 (7.84)

**Performance of a clinical malaria diagnosis and mRDT for the prediction of malaria parasitaemia among under-fives:** As expected, a clinical diagnosis of malaria had a very high sensitivity (97.0% (95%CI: 91.0-99.2)) for correctly identifying under-fives with malaria parasitaemia (Table 3). However, a clinical malaria diagnosis could correctly identify under-fives without malaria parasitaemia in only about two thirds (66.7% [95%CI: 62.3-70.8]) of under-fives. A clinical malaria diagnosis could predict presence of malaria parasites in only about a third (37.4% (95%CI: 31.6-43.5)) of under-fives, but could predict absence of malaria parasites in a very high percentage (99.1% [95%CI: 97.2-99.8]). The prevalence of malaria parasitaemia was 17.0% by microscopy and 32.8 by mRDT. The mRDT had a very high sensitivity (99.0% [95%CI: 93.9-99.9]) and specificity (80.7% [95%CI: 76.9-84.0]) for correctly identifying under-fives with and without malaria parasitaemia (Table 3). The mRDT could predict presence of malaria parasite in just about a half (51.3% [95%CI: 44.1-58.4]) of under-fives and could predict absence of malaria parasites in a very high percentage (99.75% (95%CI: 99.4-100.0)).

**Table 3:** Performance of clinical malaria diagnosis and mRDT for the prediction of malaria parasitaemia among under-fives

Performance	Clinical malaria diagnosis	mRDT findings
True positives (a)	99	101
False negatives (b)	166	96
False positives (c)	3	1
True negatives (d)	332	402
Sensitivity: a/(a+b)	97.0% [95%CI: 91.0-99.2]	99.0% [95%CI: 93.9-99.9]
Specificity: d/(c+d)	66.7% [95%CI: 62.3-70.8]	80.7% [95%CI: 76.9-84.0]
Predictive value positive: a/(a+c)	37.4% [95%CI: 31.6-43.5]	51.3% [95%CI: 44.1-58.4]
Predictive value negative: d/(b+d)	99.1% [95%CI: 97.2-99.8]	99.75% [95%CI: 99.4-100.0]

**Influence of parasitological findings on antimalarial and antibiotics prescription:** Both antimalarial drugs and antibiotics were prescribed to the under-fives attended on account of fever (Table 4). Of those receiving an antimalarial prescription, 75.0% were positive for mRDT while 39.4 were positive microscopically. Since prescription was based on mRDT findings, a quarter of the under-fives received an antimalarial despite having a negative result. If microscopic findings were to be used as the basis for antimalarial prescription, there would be a relative reduction of mRDT based antimalarial use by 47.5%. Among under-fives receiving an antibiotic prescription, about one-fifth (21.2%) were mRDT positive; however more than three quarters (78.8%) were negative for mRDT. If microscopic findings were to be used as the basis for antibiotics prescription, there would be a relative reduction of mRDT based antibiotic use by 53.3%

**Table 4:** Influence of parasitological findings on antimalarial and antibiotics prescription according to mRDT and microscopic findings

Laboratory findings	Prescription	
	Antimalarial (n =254) No (%)	Antibiotic (n = 476) No (%)
**Results of mRDT**		
Positive	191 (75.0)	101 (21.2)
Negative	63 (25.0)	375 (78.8)
**Results of microscopy**		
Positive	100 (39.4)	47 (9.9)
Negative	154 (60.6)	429 (90.1)

## Discussion

The present study show that malaria is still a problem as 22.3% of the under-fives were reported to have been treated for malaria in the last two weeks; and during the study a clinical diagnosis of malaria was entertained in 32.7% of the consultations. The observed prevalence of parasitaemia by both microscopy (17.5%) and mRDT (32.8%) at facility level is higher than the microscopic prevalence of 5.4% and 18.6% mRDT observed at the community level for Mwanza region through a recent national survey [7].

### Accuracy of Clinical Diagnosis for the prediction of malaria parasitaemia among febrile under-fives

The Tanzania national guidelines for malaria diagnosis and treatment provides that fever (history of fever or measured fever) is the entry point for suspecting malaria in an under-five but other features such as gastroenteritis (diarrhea / vomiting), pallor (palms / conjunctiva), inactivity and inability to feed together constitute a clinical malaria diagnosis [14]. The present study show that a clinical diagnosis of malaria, although not specific, has the advantage of being highly sensitive (sensitivity 97.0% [95%CI: 91.0-99.2]) for correctly identifying under-fives with malaria parasitaemia. In this situation, sensitivity is more important than specificity particularly because, although treatable, malaria is potentially fatal [15]. This emphasizes on the need for ensuring availability of guidelines and adherence to the clinical algorithm for malaria diagnosis coupled with parasitological confirmation because diagnosis based on clinical features alone has a low specificity and increases the risk of over-diagnosis especially under reduced malaria prevalence as is the current situation [16, 17].

### Accuracy of mRDT for the prediction of malaria parasitaemia among febrile under-fives

The Mwanza region had recorded a decline in malaria prevalence (by mRDT) among under-fives; from 31.4% in the year 2007/2008 to 18.6 % in the year 2011/2012 representing a relative reduction of 40.8% [6, 7]. The primary goal of this study was to examine whether the decline of malaria prevalence would have reduced the diagnostic performance of mRDT routinely used in health facilities for parasitological malaria confirmation. The present findings show that despite the decline in malaria prevalence in the study area, the mRDT still retained high sensitivity (99.0% [95%CI: 93.9-99.9]) for correctly identifying under-fives with malaria parasitaemia, and was at the level of sensitivity ≥95% recommended by WHO [18]. The mRDT also retained a high specificity (80.7% [95%CI: 76.9-84.0]) for correctly identifying under-fives without malaria parasitaemia, but this was lower than the WHO recommendation of a specificity ≥95% for an ideal mRDT.

### Influence of parasitological malaria confirmation on antimalarial and antibiotics prescriptions

Malaria rapid diagnostic tests (mRDT) have been rolled out in Tanzania following the recommendations by the WHO to adopt universal testing to confirm presence of malaria parasites before antimalarial treatment [4]. Universal testing to confirm presence of malaria parasites is envisaged to guide the prescription of an antimalarial drug to those with positive mRDT results, while those with negative mRDT results with a suspected bacterial infection would receive an antibiotic. The present findings show that of the 254 under-fives who received an antimalarial prescription, three quarters (75.0%) had positive mRDT results; only a quarter (25.0%) had negative mRDT results, implying a major adherence to test results by prescribers. Similar observations have been made in Rufiji, southeastern Tanzania whereby adherence to mRDT findings resulted in reduction of overtreatment with antimalarial drugs by more than two thirds (71.5%) conceivably resulting from the early health promotion campaigns as Rufiji was one of the districts where mRDT was first introduced as part of the national mRDT rollout [10]. Since the rollout of malaria rapid diagnostic tests for routine use in all levels of health care was envisaged as a strategy for targeting antimalarial use, in this case ACT, to only those with positive mRDT results [10], the present findings show that adherence to test results has lead to a reduction of antimalarial drug over use by three quarters as demonstrated by other studies [19, 20]. Of those receiving antibiotic prescription on suspicion of invasive bacterial disease, the large majority (90.1%) had negative mRDT results. This represents an over-treatment with antibiotics as it has been shown that even in low to moderate malaria transmission settings, invasive bacterial disease is uncommon in under-fives with non-severe illness [9]. Giving an antibiotic prescription to all febrile under-five with a negative mRDT result is not justifiable because recently it has been shown that most under-fives with fever probably have pathogens that do not require treatment with an antibiotic [21]. The fact that only about a third (32.8%) of the under-fives were parasitologically confirmed by mRDT to have malaria implies that two thirds of the of those evaluated for malaria on account of fever were suffering from a condition other than malaria. Various non-malarial infections have been found to be major causes of febrile syndromes in tropical settings but often clinically indistinguishable without confirmatory tests [21]. There is therefore a need to develop RDTs for other tropical infections for the individual case-management of non-malarial tropical infections presenting with fever [22].

## Conclusion

Findings reaffirm that a clinical malaria diagnosis is a poor marker of malarial disease that is likely to be worse under reduced malaria burden. The decline in malaria prevalence has altered the performance of mRDT to a specificity < 95% below the WHO recommendation. There was a reduction in over prescription of antimalarial by three quarters due to a high adherence to mRDT results. There was an over prescription of antibiotics as not all mRDT negatives would necessarily have an invasive bacterial disease.

### What is known about this topic

- Presumptive (clinical) diagnosis of malaria is non-specific because of the overlap of the symptoms of malaria with other infectious diseases;
- There is a decline in malaria burden in sub-Saharan Africa, including Tanzania;
- The performance of clinical diagnosis and mRDT is known to change with the prevalence of malaria.

### What this study adds

- To what extent the decline in malaria burden alters the performance of clinical diagnosis for the prediction of malaria parasitaemia under reduced malaria burden;
- To what extent the decline in malaria burden alters the performance of mRDT for the prediction of malaria parasitaemia under reduced malaria burden;
- To what extent parasitological confirmation of malaria by mRDT guides the management of febrile underfives in terms of antimalarial and antibiotic prescriptions in settings of reduced malaria burden.

## References

[1] Warrell David, Warrell DA, Gilles HM (2002). Clinical Features of Malaria. Bruce-Chwatt's Essential Malariology..

[2] D'Acremont Valerie, Lengeler Christian, Genton Blaise (2010). Reduction in the proportion of fevers associated with Plasmodium falciparum parasitaemia in Africa: a systematic review. Mal J..

[3] Seth Misago, Mdetele Daniel, Phillips Scott, Buza Joram (2015). Challenges in diagnosis of febrile illnesses in Tanzania in the era of declining malaria epidemiology. AJRes Comm..

[4] World Health Organization (2011). Universal access to malaria diagnostic testing: An operational manual..

[5] Kyabayinze DJ, Tibenderana JK, Odong GW, John B, Rwakimari JB, Counihan H (2008). Operational accuracy and comparative persistent antigenicity of HRP2 rapid diagnostic tests for Plasmodium falciparum malaria in a hyperendemic region of Uganda. Mal J..

[6] Tanzania Commission for AIDS (TACAIDS), Zanzibar AIDS Commission (ZAC) (2009). National Bureau of Statistics (NBS), Office of the Chief Government Statistician (OCGS), Macro International Inc. (2008) Tanzania HIV/AIDS and Malaria Indicator Survey 2007-08..

[7] Tanzania Commission for AIDS (TACAIDS), Zanzibar AIDS Commission (ZAC) (2013). National Bureau of Statistics (NBS), Office of the Chief Government Statistician (OCGS) ICF International. Tanzania HIV/AIDS and Malaria Indicator Survey 2011-12..

[8] Alberg Anthony, Park Wan, Hager Brant, Brock Malcolm, Diener-West Marie (2004). The Use of “Overall Accuracy” to Evaluate the Validity of Screening or Diagnostic Tests. J Gen Intern Med..

[9] Mtove George, Hendriksen Ilse, Amos Ben, Mrema Hedwiga, Mandia Victor, Manjurano Alphaxard, Muro Florida, Sykes Alma, Hildenwall Helena, Whitty Christopher, Reyburn Hugh (2011). Treatment guided by rapid diagnostic tests for malaria in Tanzanian children: safety and alternative bacterial diagnoses. Malar J..

[10] Masanja Irene, Selemani Majige, Baraka Amuri, Kajungu Dan, Khatib Rashid, Kachur Patrick, Skarbinsk Jacek (2012). Increased use of malaria rapid diagnostic tests improves targeting of anti-malarial treatment in rural Tanzania: implications for nationwide rollout of malaria rapid diagnostic tests. Mal J..

[11] World Health Organization (2013). WHO informal consultation on fever management in peripheral health care settings: a global review of evidence and practice..

[12] Mosha Jacklin, Sturrock Hugh, Greenhouse Bryan, Greenwood Brian, Sutherland Colin, Gadalla Nahla, Atwal Sharan, Drakeley Chris, Kibiki Gibson, Bousema Teun, Chandramohan Daniel, Gosling Roly (2013). Epidemiology of subpatent Plasmodium falciparum infection: implications for detection of hotspots with imperfect diagnostics. Mal J..

[13] Mazigo Humphrey, Meza Wilfred, Ambrose Emanuella, Kidenya Benson, Kweka Eliningaya (2011). Confirmed malaria cases among children under five with fever and history of fever in rural western Tanzania. BMC Res Notes..

[14] National Malaria Control Programme (2006). National Guidelines for malaria diagnosis and treatment, Ministry of Health & Social Welfare..

[15] Marsh Kevin, Snow R (1997). Host-parasite interaction and morbidity in malaria endemic areas. Phil Trans Soc Lond B..

[16] O'Meara Wendy, Mangeni Judith, Steketee Rick, Greenwood Brian (2010). Changes in the burden of malaria in sub-Saharan Africa. Lancet Infect Dis..

[17] World Health Organization (2010) (2010). Guidelines for the treatment of malaria..

[18] Murray Clinton, Gasser Robert, Magill Allan, Miller Scott (2008). Update on rapid diagnostic testing for malaria. Clin Microbiol Rev..

[19] Ishengoma Deus, Francis Filbert, Mmbando Bruno, Lusingu John, Magistrado Pamela, Alifrangis Michael, Theander Thor, Bygbjerg Ib, Lemnge Martha (2011). Accuracy of malaria rapid diagnostic tests in community studies and their impact on treatment of malaria in an area with declining malaria burden in north-eastern Tanzania. Mal J..

[20] D'Acremont Valerie, Kahama-Maro Judith, Swai Ndeniria, Mtasiwa Deo, Genton Blaise, Lengeler Christian (2011). Reduction of anti-malarial consumption after rapid diagnostic tests implementation in Dar es Salaam: a before-after and cluster randomized controlled study. Mal J..

[21] D'Acremont Valérie, Kilowoko Mary, Kyungu Esther, Sister Philipina, Sangu Willy, Kahama-Maro Judith, Lengeler Christian, Cherpillod Pascal, Kaiser Laurent, Genton Blaise (2014). Beyond malaria-causes of fever in outpatient Tanzanian children. N Engl J Med..

[22] Chappuis Francois, Alirol Emilie, D'Acremont Valerie, Bottieau Emmanuel, Yansouni Cedric (2013). Rapid diagnostic tests for non-malarial febrile illness in the tropics. Clin Microbiol Infect..

